# Structure-in-Void Quasi-Bound State in the Continuum Metasurface for Deeply Subwavelength Nanostructure Metrology

**DOI:** 10.1021/acsnano.5c03366

**Published:** 2025-09-02

**Authors:** Falco Bijloo, Arie J. den Boef, Peter M. Kraus, A. Femius Koenderink

**Affiliations:** † 530573 Advanced Research Center for Nanolithography, Science Park 106, 1098 XG Amsterdam, The Netherlands; ‡ Department of Physics of Information in Matter and Center for Nanophotonics, 55952 NWO-I Institute AMOLF, Science Park 104, 1098 XG Amsterdam, The Netherlands; § Department of Physics and Astronomy, and LaserLaB, Vrije Universiteit, 1081 HV Amsterdam, The Netherlands; ∥ ASML Netherlands B.V., 5504 DR Veldhoven, The Netherlands

**Keywords:** quasi-BIC, metasurface, metrology, nanophotonics, Fano resonance, sensor, effective index

## Abstract

Fano lineshapes associated with quasi bound-state-in-the-continuum resonances, that are supported by dielectric metasurfaces, have the advantageous properties of being extremely sensitive to minute geometrical changes in the meta-atoms. We show an approach to determine deep subwavelength feature sizes, comparable to semiconductor critical dimension metrology, by structurally infilling a void of a dielectric disk-hole metasurface design. Our simulated results show a sensitivity of 40.5 nm resonant wavelength shift for a 1 nm feature width (i.e., critical dimension) change, at an optical line width of 1.8 nm. We present both experimental and simulated results of different void infillings and attribute the spectral change of the resonance to the sensitivity to an effective index in the void of the meta-atom, which arises from the filled volume fraction and the material boundaries orientation relative to the local polarization. Treating our metasurface as an effective index sensor, the sensitivity is 262 nm·RIU^–1^ and the figure of merit is 146 RIU^–1^, which underlies the pronounced resonant wavelength shift driven by similarly large changes in the effective index caused by extremely tiny critical dimension variations. This approach could impact critical dimension measurements in semiconductor metrology, as it works at the high throughput of optical measurements while performing at the high resolution of scanning electron microscopy.

## Graphical abstract

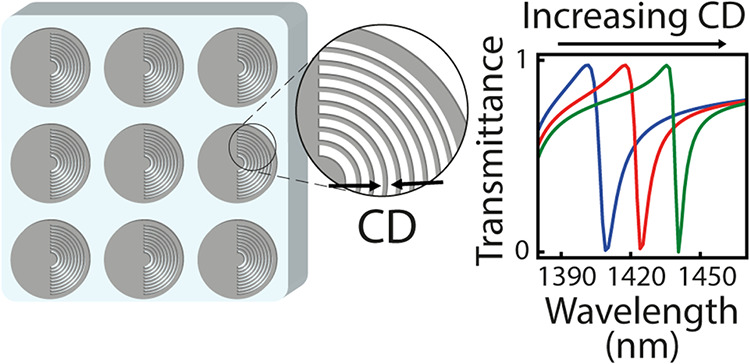

Fano resonances in optical dielectric metasurfaces have uniquely advantageous properties for manipulating optical near- and far-field responses. Fano resonance lineshapes generally arise from the interference between a broad continuum of states (such as a broad scattering resonance or background radiation) and a narrow discrete resonance (such as a high-Q Mie mode).^1–3^ The quality factor *Q* and overall line shape can be exquisitely controlled by, for instance, combining broad dielectric Mie resonances to shape the continuum,^4^ with exquisite tailoring of the radiative damping of the narrow resonance by using, e.g., multipole resonances,^5,6^ guided mode resonances,^7,8^ or controlled symmetry breaking.^9–11^ As Fano resonances carry a strong near-field, a nontrivial phase response, and often also wave vector selectivity, they allow for the shaping and manipulation of wavefronts,^12,13^ efficient harmonic generation,^14,15^ very sensitive refractive index sensors,^16–19^ and other applications.^20–22^ An efficient strategy for generating strong resonances has proven to be the engineering of quasi-bound states in the continuum (quasi-BIC) metasurfaces.^23–26^ The main principle of a BIC is the vanishing coupling between a discrete resonant mode and all radiation channels, called the continuum, which is generally the background radiation. The most common approach starts with a symmetry-protected BIC metasurface, using a periodic design that supports a Bloch mode prohibited from radiating due to symmetry constraints. Building on such a dark mode – which lacks radiation damping and thus has a nominally infinite *Q* – resonances can be unveiled by introducing a slight asymmetry in the unit cell or superlattice. This asymmetry opens a radiation channel, allowing the mode to radiate.^27^ As the original BIC becomes leaky, it is referred to as a quasi-BIC.^9^ It is well established that by tuning the geometric asymmetry, one can tailor the quality factor and resonant wavelength at will.^26^ By choosing a specific geometric asymmetry one could, for example, use a quasi-BIC metasurface as a sensitive gas-sensor, by tracking the resonant wavelength as a function of surrounding refractive index.^28^ These examples show the strong responsivity of quasi-BIC metasurface resonances to tiny perturbations in the nanostructure or surrounding medium.

Nanolithography techniques can achieve resolutions down to tens of nanometers in scientific settings, (electron beam lithography), while the semiconductor industry is pushing the limits below 5 nm, with sub-nm accuracy using extreme ultraviolet technology.^29–32^ Metrology, the science of measuring and determining specific geometrical or dimensional parameters, plays an essential role in the semiconductor industry.^30^ Commercial lithography demands metrology techniques that match the accuracy of the lithography process itself. Chip fabrication involves numerous sequential steps, each requiring precise dimensioning and alignment.^29,33,34^ Manufacturing efficiency — in terms of yield, and thereby resource utilization — crucially depends on the ability to perform exquisite quality control at every stage. In the semiconductor industry, this quality control is achieved through metrology. The economic impact of improved yield through faster and more accurate metrology is immense: with a global annual revenue of $600 billion in lithography, even a 1% improvement in yield represents a value of $6 billion.^35^ A wide range of structural parameters are of interest for metrology, including individual layer thicknesses, relative alignment of different layers (so-called interlayer overlay), feature sidewall angles, line-edge roughness, and more.^34^ Among these, one of the most fundamental parameters is the so-called critical dimension (CD), which represents the width of the smallest feature size of a nanostructure. CD measurements at the highest spatial resolution can be achieved with scanning electron microscopy (SEM). To employ it, wafers need to be taken out of a production line. While CD-SEM provides excellent accuracy, it suffers from low throughput, potential beam-induced damage, and SEM-specific error sources such as drift and inaccurate beam placement due to charging effects.^30,34,36^ There is hence a large role for optical methods. Optical methods are noninvasive and nondestructive, and the fact that they are capable of delivering very high throughput (∼0.1 s^34^) means that they are routinely applied in the manufacturing loop, screening every wafer either after resist development or etching. However, optical methods are fundamentally constrained by the diffraction limit. The challenge is to extract values for geometrical parameters with (sub)-single digit nanometer accuracy while using visible wavelengths.

Optical metrology is typically performed not on actual devices but on dedicated targets that are specifically designed for efficient, fast, and accurate measurements. The target design includes important similar features of the actual functional device, without fully replicating it, to extract the most useful information. These metrology targets are patterned in the same litho-step as the functional integrated circuits and are placed in the scribe lanes of a wafer, spatially separated from and located between the devices. By choosing the right design, position and orientation, the important metrology information on the targets can be reliably extrapolated to the device features.^34^ Some targets are designed to extract multiple metrology parameters, while others are tailored for a single task. Depending on the application, a single wafer can include dozens to hundreds of metrology targets for measuring alignment- and overlay errors, CD (variations) and surface roughness, among others. The most common optical approach for determining CD relies on spectral measurements of specially designed periodic nanostructure scatterometry targets, using a library-based approach to reconstruct grating parameters. These techniques require prior knowledge of the grating’s periodicity and approximate structural parameters. Indeed, the crucial distinction between general imaging and reconstruction problems is that metrology seeks to accurately determine the value of a single parameter given as complete knowledge as possible of the sample geometry. It is this crucial distinction that enables beating the diffraction limit by several orders of magnitude. For instance, diffraction based metrology (based on measuring grating diffraction efficiency) is widely used in metrology to determine overlay errors (i.e., relative interlayer alignment with ASML Yieldstar^37^) with single digit nanometer accuracy. Diffraction based scatterometry is also effective—even with very low optical contrast—for CD measurements for features above tens of nanometers, but accurately measuring CD at the single-digit nanometer scale with high throughput remains an open challenge. To address the growing demands of modern semiconductor metrology, new solutions are required that reach both the high throughput capabilities of scatterometry and the sub-nm resolution provided by SEM. These requirements ask for the development of optical metrology techniques that give strong transmission or reflection contrast to reach fast and accurate readout, while maintaining a minimal wafer footprint that is suitable for the integration in advanced metrology workflows. This work is motivated by the notion that metasurface designs can present competitive optical scattering targets for metrology. For measurements on “telltale" scattering targets to be relevant, their design must meet a set of demands: (1) the targets must include feature sizes that are comparable to those in the device, (2) they should occupy minimal footprint to preserve valuable wafer real estate, and (3) they should allow for rapid measurements to support high-throughput processes. Meeting these criteria directly impacts the yield by reducing the number of faulty devices, increasing the available area for functional integrated circuits, and enabling measurements within a shorter time frame.

In this Article, we present a method for optically determining the CD of dielectric nanostructure patterns with deeply subdiffractive periodicity and single-digit nanometer widths, achieving subnanometer sensitivity based on the concept of quasi-BIC metasurfaces. The method is based on studying the spectral response of the metasurfaces composed of dielectric meta-atoms featuring an asymmetrically placed semicircular void, which induces a quasi-BIC, while the void itself is embedded with the structures under test (satisfying requirement (1) above). We report both simulations and experiments, reaching a simulated sensitivity of 40.5 nm resonant wavelength shift per 1 nm increase in CD at 7 nm feature size. This theory is supported by experiments on prototype samples (experiments at 1.5 μm wavelength, at larger CD values). We attribute the observed shifts in the Fano resonance to the exceptional refractive index sensitivity of quasi-BIC metasurfaces. Structural changes of the subwavelength nanostructures, such as variations in width, modify the effective index of the meta-atom structure-in-void, leading to a pronounced resonance shift. We substantiate this understanding by analyzing Fano resonance shifts for structure-in-void metasurfaces with meta-atoms that consist of different shapes, where the subwavelength patterns are aligned either along or perpendicular to the local polarization. By mapping the data onto the effective index using a polarization-dependent effective index model, we demonstrate that all sensitivity curves converge onto a single master curve. Our findings introduce a unique approach for detecting ultrasmall feature variations in deeply subwavelength gratings embedded within dielectric metasurfaces. Although the mechanism that we identify is akin to reported sensing of refractive index changes in gases and liquids by BIC metasurfaces, the performance metrics are different: the standard approach to BIC index sensing is to work at very high quality factors *Q*, in turn requiring semi-infinite spatial extent. Instead, metrology targets must occupy only a small spatial footprint (requirement (2) listed above). Therefore, we developed a method that works at low quality factor *Q*, and equivalently poor angular resolution. Despite low *Q*, in our approach small variations in CD produce pronounced shifts in the reflection or transmission spectrum. The approach is fully compatible with existing diffraction-based metrology workflows at very high speeds (requirement (3) above) and offers immediate applicability in advanced semiconductor manufacturing. While conventional methods rely on scatterometry on non-resonant subdiffractive gratings to reconstruct grating parameters, our approach harnesses the strong electric field confinement of quasi-BIC metasurface resonances. In essence, current metrology targets used in scatterometry can be seamlessly integrated into a structure-in-void metasurface platform to enhance optical contrast from just a few percent to several tens of percent. This improvement enables higher yield through faster measurements, and more accurate estimation of critical dimensions down to the single-digit nanometer scale.

## Results and Discussion

Figure 1 shows the main concept of our metrology proposition, which is based on a metasurface comprising high-index disks with an asymmetrically placed structure-in-void illuminated by white light to measure a transmission spectrum. It is well-known that such a disk system with a symmetrically placed hole can support a symmetry-protected BIC mode, which becomes optically accessible in the far field, and turns into a quasi-BIC, upon introducing an asymmetry by off-center placement of the hole.^25,27,38,39^ Similar disk-hole structures have been proposed for dynamic nonlinear image tuning^38^ – where the dark mode is attributed to a magnetic dipole resonance with out-of-plane magnetic moment – and cube-hole structures for a polarization-independent sensor.^40^ Strong resonances can occur in a variety of similar structures with asymmetrically placed air holes, involving magnetic dipole resonances, electric quadrupole resonances, and toroidal dipole responses.^24^ Experimentally, for such structures, quality factors up to ∼5·10^3^ can be obtained at λ ∼1500 nm, tunable by the placement and size of the hole. In our work, the goal is not to maximize *Q* by minimizing size and optimizing placement of the hole, as metrology targets requires functionality at the small footprint of only a few unit-cells, which inherently reduces the quality factor. We rather use the hole area as the metrology target region where we embed a deeply subwavelength grating with a known periodicity, similar to current subdiffractive metrology gratings, to measure the unknown CD. Therefore, we focus on designs in which the hole is semicircular and extends over nearly half of one side of the disk, showing that the prototype already functions well at relatively low *Q*. The nanostructures under test are placed in the void, forming what we refer to as the “structure-in-void” metrology sensor. Throughout this article, any references to “placing,” “filling,” or “embedding” structures refer strictly to the design stage of the metasurface geometry. All features of the design are fabricated in a single lithography step. These terms do not imply any physical postprocessing steps such as postfabrication mechanical insertion, a second lithography and lift-off step, or material infiltration.

**Figure 1:**
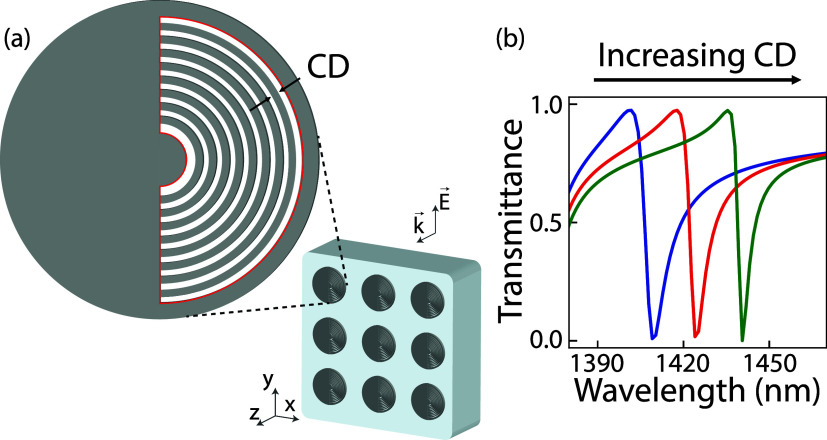
(a) Schematic illustrating the core concept. We illuminate a quasi-BIC metasurface – composed of thin silicon disks (height *h* = 75 nm, radius *r_d_* = 400 nm, pitch *p* = 995 nm) with an asymmetrically placed structure-in-void– on a quartz substrate using white light to measure the transmission spectrum. The void area is illustrated by a red solid border, and the amount of concentric rings in the void is *N* = 8. The spectral response is highly sensitive to tiny geometrical variations in the structure-in-void’s grating width, or CD, which modulates the effective index. (b) Simulated transmittance spectra for CD = 13.3 nm (blue), 16.6 nm (red) and 19.9 nm (green), indicating a clear redshift of the Fano resonance as per increasing CD.

In the field of quasi-BIC metasurfaces one usually defines an asymmetry parameter α according to meta-atom geometry, for instance defining α = 1 – (*r_d_* – *d*)/*r*_*d*_, where *d* is the distance of the hole from the center of the disk of radius *r_d_*. Since in our case, we are not interested in the placement or size of the hole per se, but in measuring the structure-in-void parameters, we instead define α = 1 – *FF*, where *FF* is the nanostructure fill factor, determined by the fraction of volume of the embedded nanostructure in the meta-atom’s void relative to an empty void *FF* = *V*_Si_/*V*_void_. The fill factor *FF* ranges from 0 (empty void) to 1 (completely filled void, symmetric disk, resulting in α = 0). In the example case of Figure 1a, the void is filled with eight azimuthally oriented lines (concentric grating) of ca. CD ≃ 16.6 nm, at a period of 33.1 nm (α = 0.5). Figure 1b shows three calculated transmittance spectra for CD = 13.3 nm, 16.6 and 19.9 nm, (*FF* = 0.4, 0.5 and 0.6) where a clear red shift of the Fano resonance is visible for increasing CD.

Figure 2a presents finite element simulations (COMSOL Multiphysics 5.2) for the normal-incidence transmittance through a structured void metasurface with *N* = 8 lines, where we increase the thickness of the concentric rings to obtain fill factors from *FF* = 0 to 1. We solve for the system assuming polycrystalline silicon meta-atoms in air on glass (*n*_air_ = 1, *n*_glass_ = 1.44, *n*_Si_ = 3.45), placed in an infinite square array of pitch *p* = 955 nm. A detailed description of the simulation is presented in the Methods section. The meta-atoms are illuminated from the glass side with polarization along the vertical axis (*y*-axis in Figure 1a, perpendicular to the horizontally placed asymmetry), inducing an asymmetric electric near-field on either side of the meta-atom. This asymmetry drives a circulating electric current that is associated with an out-of-plane magnetic Mie dipole, which is visualized by a ring in the normalized electric field (inset of Figure 2c). The transmittance is calculated as the energy flow from input port (in the glass) to output port (air). At *FF* = 1 (symmetric disk, completely filled void) the metasurface supports a vanishing symmetry protected BIC, however by introducing an asymmetry (reducing the *FF* to 0.9), a sharp resonance occurs near 1500 nm wavelength of *Q* = 3·10^3^. Such high *Q*-values are typical for quasi-BIC resonances with very low asymmetry values^23^. The quasi-BIC resonance blue shifts and significantly broadens upon increasing the asymmetry (decreasing *FF*), reaching a wavelength of 1380 nm and a *Q* = 200 at zero fill factor. This behavior is archetypical for symmetry-protected quasi-BIC metasurfaces. A second spectral feature is found around 1370 nm, which does not show a vanishing line width as the asymmetry is removed. This feature is associated with a Rayleigh anomaly (RA), where the meta-atom bright mode mediates grating diffraction into glass.

**Figure 2:**
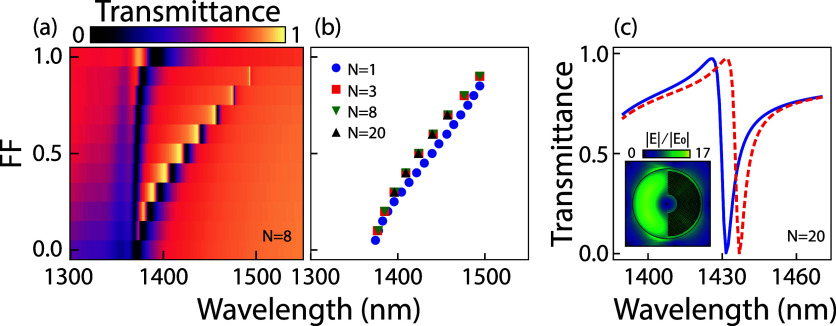
Simulated spectral response of a structure-in-void metasurface, composed of meta-atoms of disks with an embedded concentric grating in the asymmetrically placed void (illustrated in Figure 1a), as a function of fill factor. The variable *N* denotes the number of concentric rings in the grating. (a) Transmittance spectra for *N* = 8, for fill factor *FF* on the *y*-axis ranging from 0 (fully asymmetric/empty void) to 1 (no asymmetry/completely filled void), corresponding to CD ranging from 0 to 33.1 nm. A blue-shift and broadening of the asymmetric Fano line shape is clearly visible for decreasing *FF*. A somewhat broader resonance is relatively wavelength independent and present around 1370 nm. (b) Fano-dip tracking for *N* = 20, 8, 3 and 1, plotted as a function of *FF* (on the *y*-axis), in which it is clear that similar behavior is found for all *N*. This result enables us to scale up the nanostructure CD for experimental purposes, demonstrating proof-of-principle measurements that operate similarly for very small CDs in industrial applications. (c) Transmittance spectra for *N* = 20, for two fill factors *FF* = 0.55 (blue solid) and 0.56 (red dashed) corresponding to CDs of 7.29 and 7.42 nm respectively, which shows a 5.4 nm wavelength shift of the Fano resonance for a 0.13 nm increase in CD. At this wavelength the optical line width is 1.8 nm, with *Q* = 805. The inset shows the simulated electric near-field |*E*|, normalized to the incoming electric field |*E*_0_|, for a single meta-atom with *N* = 20 (*FF* = 0.56) at the Fano dip of 1437 nm.

We focus on the quasi-BIC and examine the transmittance for structure-in-void metasurfaces for meta-atoms with varying coarseness of the azimuthal line pattern *N* = 1, 3, 8, and 20 concentric grating lines. Note that for each value of *N*, the periodicity of the nanostructure is well-defined, as it is given by the void radius divided by the number of concentric grating lines. This results in a unique mapping of *FF* to CD. The embedded nanostructure CD ranges from 0 nm at *FF* = 0 to 265 nm (*N* = 1), 88.3 nm (*N* = 3), 33.1 nm (*N* = 8), and 13.3 nm (*N* = 20) at *FF* = 1. To underline the generalization of the resonance shift behavior as a sensing mechanism to CD variations, we track the Fano dip as a function of *FF* in Figure 2b. Similar behavior for *N* = 3, 8, and 20 is found, while the *N* = 1 case shows a small deviation from the common behavior, though being qualitatively similar. On basis of this simulation result, we hypothesize that the Fano-resonance is primarily sensitive to the fill factor of the nanostructure in the void, which in the spirit of homogenization theory is equivalent to measuring effective index of the void volume. For prototype experiments in this work we test the concept for modest numbers of lines, compatible with the CD values that can be achieved in standard academic electron beam lithography processes. On basis of the numerical simulations we argue that the concept extrapolates to large *N*, i.e., to the very small CD that can not be achieved in an academic cleanroom, and that may be achieved only in industrial settings. This scaling allows us to experimentally validate the concept while demonstrating proof-of-principle measurements that function similarly for very small CDs in industrial applications. As industry has a need for “at-resolution” metrology, i.e., metrology on structures with the same dimensions as device structures, this is an essential capability. Figure 2c presents simulated transmittance results for a metasurface with *N* = 20 azimuthal lines for *FF* = 0.55 and 0.56, which is on par with industrial resolution limits. The small change in fill factor corresponds to a change in CD of 0.13 nm, from 7.29 to 7.42 nm. This minute geometrical change causes a resonance shift of ca. 5.4 nm in wavelength. Given the quality factor *Q* = 805 at this fill factor, already a full optical line width shift of 1.8 nm is reached upon 0.05 nm CD variation, which is smaller than the lattice spacing in crystalline silicon. This strong fill factor dependence evidence an extremely high sensitivity for the readout of geometrical variations, even for much lower quality factors due to finite-size arrays with a footprint of <10 μm, that would be practically considered in metrology applications. The Supporting Information (SI) reports on 10 × 10 (and 20 × 20) unit-cells simulations, which reveal *Q* ≃ 25 (*Q* ≃ 89) at a modest *FF* = 0.55, which still corresponds to an optical line width shift at single (sub) nm-sized CD variations.

We fabricated metasurfaces in 75 nm thick polycrystalline silicon, evaporated on fused quartz, via a standard electron beam lithography procedure. This height was chosen to approach ultrathin layers that are used in industry, while maintaining enough material to excite the out-of-plane MD associated with the quasi-BIC resonance. A detailed description of the nanofabrication procedure is found in the Methods section. The metasurfaces consist of meta-atoms placed in square arrays (995 nm pitch) with disks of radius *r*_*d*_ = 400 nm and a semicircular hole that covers almost half one side of the disk, stretching from *r*_1_ = 100 nm to *r*_2_ = 365 nm. We created a variety of embedded nanostructure patterns including azimuthal line gratings, radial spokes and dot patterns, where we vary the fill factor and the number of elements *N* within the constraints of the e-beam lithography resist (step size 5 nm and minimum gap size/CD 20 nm).

Figure 3a shows a scanning electron micrograph (SEM) image for a metasurface where meta-atoms have *N* = 1 azimuthal line in the void. The azimuthal line is centered at a radius *r* = 230 nm from the metasurface origin, and ranges in width from 64 nm (*FF* = 0.24) to 249 nm (almost filling the entire hole, *FF* = 0.94). Figure 3b presents transmittance spectra (*x*-axis) as a function of *FF* (*y*-axis). Transmission measurements are performed in a home-built microscope setup that loosely focuses white light onto the metasurface from the glass side (*f* = 30 mm, NA ∼ 0.05), while collecting many angles on the air side with an objective (NA 0.9, 100×, Nikon, CFI Plan Apo BD) and projecting the image onto a multimode fiber (core size 100 μm) that feeds into a Fourier transform optical spectrum analyzer (Thorlabs, OSA202C). Throughout this work, the *FF* is determined from SEM images by image analysis, using a thresholding procedure described in the SI. Incident white light is polarized along the vertical axis (*y*-axis in Figure 1), allowing excitation of the quasi-BIC mode. Similar Fano line shapes between 1380 and 1500 nm as in the simulation are found. A broad spectral feature around 1360 nm is found at all asymmetries that is associated with the Rayleigh anomaly, while to the red of the Rayleigh anomaly the quasi-BIC is observed. Similar to the simulation, the quasi-BIC broadens and blue shifts with increasing asymmetry (decreasing *FF*). Features appear much broader than in the simulation, as in the experiment we loosely focus on the sample, causing an averaging over a cone of excitation angles, while additionally introducing finite footprint that fundamentally constraints quality factor (see SI for 20 × 20 unit-cell simulations, that reveal a simulated *Q* ≃ 89, which is close to the measured *Q*). Furthermore, since our academic e-beam lithography tools inherently introduce CD variations within a metasurface, all resonances are smeared out across the range of resonances that occur due to the variations in the fabricated CDs.^41^ Spectral fringes of ca. 1.3 nm are observable in the experiment, which are likely caused by a Fabry–Perot effect from the quartz substrate, that is not present in the simulation.

**Figure 3:**
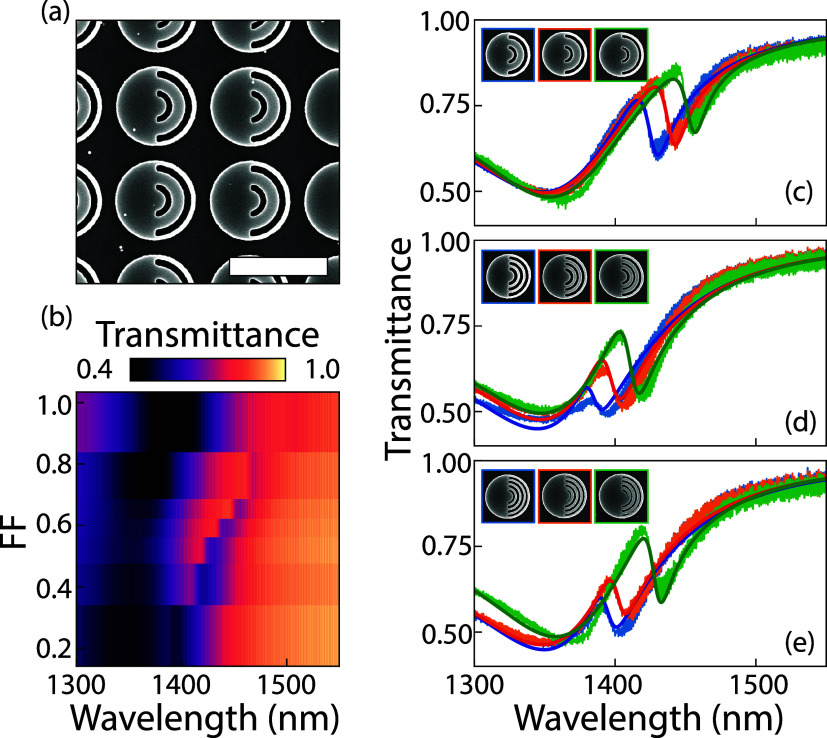
Experimental realization of a structure-in-void metasurface with *N* = 1, 2, and 3 azimuthal lines. (a) SEM image for *N* = 1, *FF* = 0.51 (scale bar 1 μm). Metasurface parameters are height *h* = 75 nm, *r*_disk_ = 400 nm, with a void that covers almost half of disk reaching from *r*_1_ = 100 nm to *r*_2_ = 365 nm, with a single azimuthal line embedded in the void that is centered at *r* = 230 nm and has a width of CD = 130 nm. (b) Transmittance spectra for *N* = 1 azimuthal line nanostructured void metasurfaces with *FF* on the *y*-axis ranging from 0.24 to 0.94. (c) Spectra for *FF* = 0.51 (blue), 0.61 (orange) and 0.63 (green), showing a clear shift in resonant wavelength, accompanied by insets of SEM images of their individual meta-atoms, bordered by corresponding colors. The solid line shows a Fano line shape fit. (d) Spectra for *N* = 2 azimuthal lines structure-in-void metasurfaces with *FF* = 0.31 (blue), 0.40 (orange) and 0.48 (green), showing a similar trend, with the inset showing SEM images of individual meta-atoms. (e) Spectra for *N* = 3 azimuthal lines, *FF* = 0.40 (blue), 0.52 (orange) and 0.60 (green).

Three transmittance spectra for estimated *FF* = 0.51, 0.61, and 0.63 are plotted in Figure 3c, accompanied by insets of SEM images of their corresponding individual meta-atoms. A shift of 27.5 nm in resonance wavelength is found for a FF change of 0.12, which corresponds to 229 nm/*FF* change over the region around *FF* = 0.55, or Δ*λ*/Δ*d* = 229 [nm]/265 [nm] = 0.86 nm resonant wavelength shift for 1 nm change in CD of the single azimuthal line (while *Q* = 112, so the optical line width of the resonance is 13 nm). Assuming identical sensitivity to fill factor for higher *N* one can estimate the sensitivity for the *N* = 20 case, where Δ*d* = 13.1 nm, meaning Δ*λ*/Δ*d* = 17.4 nm resonant wavelength shift for a 1 nm change in thickness. This number is a factor two lower than the COMSOL prediction, which may be caused by rough estimation of experimental *FF*. Figure 3d presents transmittance spectra for a metasurface with *N* = 2 instead of just *N* = 1 azimuthal lines embedded in the void, accompanied by insets of SEM images of corresponding unit cells. The estimated fill factors are *FF* = 0.31, 0.40, 0.48. Similar transmittance spectra are plotted in Figure 3e, where the amount of azimuthal lines in the meta-atom void is *N* = 3, and the estimated *FF* = 0.40, 0.52, and 0.60. The measured sensitivity of this *N* = 3 metasurface is Δ*λ*/Δ*d* = 3.6 nm resonant wavelength shift per 1 nm CD increase, which means a shift of one optical line width of 10.3 nm is reached with a CD increase of 2.87 nm. Depending on the signal-to-noise ratio of a measurement protocol, even with this relatively simple design, sub-nm CD variations are within reach.

To investigate our hypothesis that *FF*, or more precisely, effective index, is the dominant contributing factor that determines the shift in the resonant response, we fabricated a plethora of different structure-in-void metasurface designs. Figure 4 presents both simulated and experimental results of the structural design study. Figure 4a shows a finite element simulation of the electric near field ( $\left|E\right|=\sqrt{E_{x}^{2}+E_{y}^{2}+E_{z}^{2}}$ ) normalized to the incoming electric field |*E*_0_| at *P* = 1W ( $\left|E_{0}\right|=\sqrt{\frac{2P}{c\epsilon_{0}p^{2}}}$ , with *p* the pitch) for a wavelength at the minimum of the Fano resonance in transmission for a reference structure, namely a disk-void metasurface where the void volume is filled with a homogeneous medium of refractive index *n*. This reference case is used to compare the effective index generated by structural infilling with line, spoke and dot patterns. The transmittance spectra (*x*-axis) for *FF* 0 to 1 (*y*-axis) are presented in Figure 4b, where *FF* is directly converted to effective refractive index by *n* = *n*_air_ + *FF* (*n*_Si_ – *n*_air_), ranging from *n* = 1 to 3.45 respectively.

**Figure 4:**
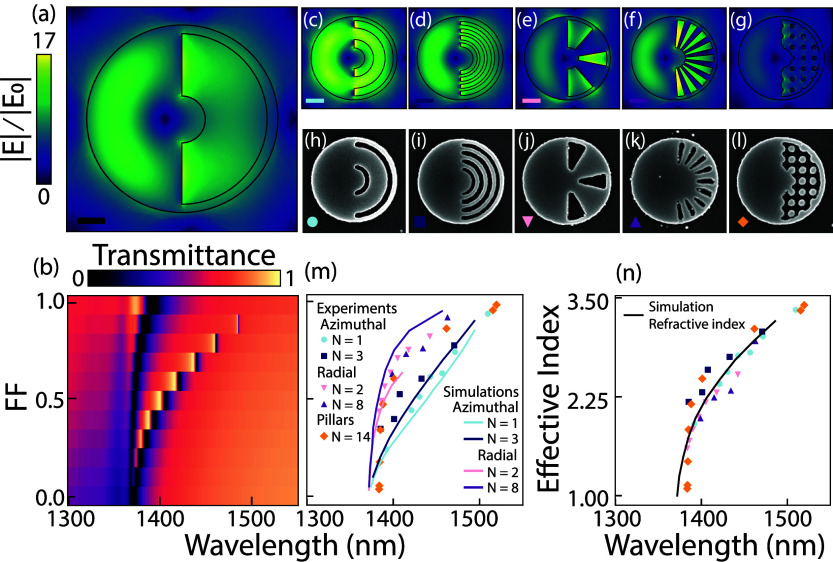
Structural design study of deeply subwavelength structure-in-void metasurfaces with near-field simulations, SEM images, and experimental Fano dip analysis. (a) Simulated electric near-field distribution for a single unit cell in a periodic lattice, with a void that is filled with refractive index *n* = 1.98, normalized to the incoming electric field |*E*_0_|, and (b) the calculated transmittance spectra for *n* = *n*_air_ (*FF* = 0) to *n* = *n*_Si_ (*FF* = 1). (c–g) Simulated normalized near-field distributions, at the Fano resonance minimum wavelength, for metasurfaces that feature designs of nanostructures embedded in the void: 1 azimuthal line (c), 3 azimuthal lines (d), 2 radial spokes (e), 8 radial spokes (f) and pillars (g), with colorscale similar to (a). (h–l) SEM images of unit-cells, with the same designs as the simulated unit-cells. (m) Fano dip track of the metasurface transmission as a function of *FF*. Experimentally extracted Fano dip wavelengths are plotted as solid markers, with colors matching the marker in the left-bottom corner in the corresponding SEM images. Simulated Fano dip tracks are plotted as solid lines, with colors corresponding to the line in the near-field simulation images. (n) Fano dip track as a function of calculated effective index, revealing that most data align along a single curve, with the Fano dip track of the refractive index void filled metasurface of (a), plotted as the solid black curve. This indicates that the metasurfaces function as effective index sensors, detecting collective structural changes in CD that are sensed through effective index variations.

In the structural design study presented in Figure 4 we highlight five different designs: (c,h) Azimuthal *N* = 1 (concentric grating), (d,i) Azimuthal *N* = 3, (d,j) Radial *N* = 2 (spokes), (f,k) Radial *N* = 8 and (g,l) Pillars *N* = 14 (dots). These designs were chosen to probe the difference between small and large *N*, and also to understand the influence of the orientation of the features relative to the electric field of the dark mode. The Fano-resonance is attributed to the coupling to an out-of-plane magnetic dipole moment, which has an azimuthally oriented in-plane electric field circling the meta-atom center. Consequently, the near field is oriented along the material boundaries for azimuthal geometric features, whereas in the radial case, the polarization is orthogonal to the boundaries. The pillar case provides a mixture of parallel and perpendicular polarizations. One might expect different sensitivities in these three cases owing to the large difference in field boundary conditions, i.e., owing to whether the perturbation is associated with a jump or with continuity in the electric field. In effective medium theory this difference expresses in different mixing formulas depending on the field orientation.^42–44^ Figure 4c–g shows simulated electric near-fields at the Fano minimum frequency, normalized to the incoming electric field |*E*_0_|, for the 5 highlighted designs. The fill factors are *FF* = 0.4, 0.4, 0.43, 0.55, 0.4. The distinction in boundary condition is immediately evident: electric field jumps in the radial metasurfaces in e and f are clear, whereas the electric field is continuous for the azimuthal line patterns in c and d. Moreover, the azimuthal (radial) designs allow a stronger enhancement of the field inside the silicon (gaps), and the pillars show an overall less enhancement. Figure 4h,l shows SEM images of individual fabricated meta-atoms for all design cases, at fill factors *FF* = 0.63, 0.4, 0.56, 0.73, 0.47.

Spectral transmission measurements were performed on all metasurface designs and at many fill factors (all spectra are reported in the SI), from which we extract the wavelength of the Fano resonance minimum (Figure 4m). Experimental results are plotted as solid markers alongside simulated results as solid lines (plot colors match the color coding in the left bottom corner of the SEM images and electric near field plots). All structural designs show a large sensitivity to geometrical change. At the same time one observes that different design families have very different responsivity curves: notable, the azimuthal designs have a similar responsivity across the entire FF-range, while the radial designs show high responsivity for large *FF* (above *FF* ∼ 0.55, steepest part of the curves), and poorer sensitivity at small *FF*. The pillar structures response lies between these extremes. This result evidence that not simply the geometrical fill factor dominates the resonance shift. We attribute this difference to the fact that the simple mixing rule *n* = *n*_air_ + *FF* (*n*_Si_ – *n*_air_) for the effective index *n* as a function of fill factor *FF* is not accurate, and needs to be refined in dependence of the family of patterns. Indeed the same *FF* can correspond to quite different effective index values depending on the orientation of structural boundaries relative to the electric field, as is well-known from the study of effective medium mixing rules for high-index contrast media (silicon and air) such as stratified stacks, nanopillar arrays and nanopore arrays with boundaries mostly following or perpendicular to the electric field. Such mixing rules have been studied extensively in the context of two-dimensional photonic crystals^42,44,45^ and effective medium theory of stratified media.^46,47^ For such systems one can single out TE-polarized propagation (*E*-field along material boundaries) and TM-polarized propagation (*H*-field along boundaries, meaning the *E*-field is not continuous), and reported mixing rules are

1 $$n_{\mathrm{eff}}^{E}={\left[\varepsilon_{a}+\mathrm{FF}\left(\varepsilon_{b}-\varepsilon_{a}\right)\right]}^{1/2}$$

2 $$n_{\mathrm{eff}}^{H}=\varepsilon_{a}{\left[\frac{\varepsilon_{a}+\left(\frac{1+2\mathrm{FF}}{3}\right)\left(\varepsilon_{b}-\varepsilon_{a}\right)}{\varepsilon_{a}+\left(\frac{1-\mathrm{FF}}{3}\right)\left(\varepsilon_{b}-\varepsilon_{a}\right)}\right]}^{1/2}$$

where *ε*_*a*_ (*ε*_*b*_) is the permittivity of the two media, in our case air (1^2^) and silicon (3.45^2^), respectively. In the structure-in-void metasurfaces, polarization is given by the direction of the circulating electric field associated with the magnetic Mie dipole. The anisotropic structuring causes form birefringence, so that the effective index depends on the field alignment relative to the structure boundaries. Whether the electric field is oriented parallel or perpendicular to the material boundaries depends on the specific meta-atom design. We apply the effective medium mixing rules accordingly, selecting eq 1 to describe the effective index that is generated by azimuthal structures (where the electric field is mostly parallel to the boundaries) and eq 2 for radial structures (where the field is predominantly perpendicular). This allows to replot the Fano resonant wavelength shift as a function of the effective index, presented in Figure 4n. In the case of pillars we chose the effective index to be described as an average refractive index according to the *FF* as

3 $$n_{\mathrm{eff}}=\varepsilon_{a}^{1/2}+\mathrm{FF}\left(\varepsilon_{b}^{1/2}-\varepsilon_{a}^{1/2}\right)$$

which takes values in between *n*_eff_^*E*^ and *n*_eff_^*H*^. For reference, we have also calculated the wavelength shift for the case of filling the void volume with a homogeneous medium of index *n*, according to the same average index eq 3. We observe that as a function of effective index all tuning curves are much closer, essentially overlapping to within the error bars in our experiments. From this analysis we can draw two distinct conclusions.

First, the mechanism of sensing is essentially that the Fano resonance is ultrasensitive to the effective refractive index of the structure-in-void *n*_eff_, which arises from the deeply subwavelength nanostructure embedded in the void. To make our sensing scheme comparable to metasurface refractive index sensors that are used in i.e., biosensing^19,48,49^ we examine the common parameter for sensitivity, which is wavelength shift per refractive index unit (RIU) change. For the structures at hand the sensitivity *S* = Δ*λ*/RIU evaluates as 262 nm/RIU for the simulated case of *N* = 20 azimuthal lines 2. With an optical resonance line width of 1.8 nm, this translates into a figure of merit (FOM, comparing the spectral shift per RIU given by *S* to the optical line width) of FOM = 146, which is comparable to reported optical sensors based on metasurfaces,^18,48,50^ but not outperforming recent works.^28^ The fact that our prototype operates at relatively low *Q* (thus FOM), yet resolves filling factor variations below 0.1 (equivalent to a 1.3 nm CD change for a 7 nm nominal CD with *N* = 20 grating lines at a 13.5 nm pitch) demonstrates the strength of our design as a metrology platform. It effectively integrates previously unused resonances with established optical metrology techniques based on scatterometry of subwavelength gratings. While comparisons to metasurface refractive index sensors may provide useful context within the broader landscape of recent studies, our aim is not to compete on sensitivity benchmarks, but to introduce a new design strategy tailored to the demands of advanced wafer metrology.

Second, the local polarization dependence of the mixing rules provides some room to optimize sensitivity, or to gain information about the anisotropy and orientation of the embedded nanostructure. Aside from possible opportunities to sense shape anisotropies in lithography (comparing CD in different dimensions), the orientation dependence also provides a route to optimize sensitivity to CD, depending on the *FF* regime of interest. For instance, for *FF* > 0.6, sensitivities are twice higher for radially oriented structures, while at the same time the optical line width becomes very narrow.

Finally, we note that the experimental data and simulated data do not perfectly collapse on the tuning curve for homogeneous void volume filling. We attribute this deviation to the fact that we assumed mixing rules that were derived for bulk electromagnetic composite media. In contrast, the nanostructures in the void at hand are themselves deeply subwavelength in diameter and height (ca. λ/7 in the *xy*-plane, and λ/20 in thickness), and in reality the modes in our structured void metasurface have boundaries both parallel and perpendicular to the electric field in all cases. We note that although the model provides a mechanistic understanding, it is not a substitute for full wave simulations. This furthermore holds for practical metrology applications, where the effective index approach could serve an insightful heuristic model, but cannot replace data fitting to rigorous spectral libraries.

## Conclusions

In summary, we presented simulations and experiments demonstrating structure-in-void quasi-BIC metasurfaces as promising sensors for critical dimensions in extremely deep-subwavelength patterns, such as encountered in semiconductor lithography. The use of the void in quasi-BIC metasurfaces as a container for metrology to embed deep subwavelength structured systems has, to our knowledge, not been proposed before. Previous works have proposed quasi-BIC metasurfaces as highly sensitive refractive index sensors,^17,19,28,51,52^ with envisioned applications in IR spectroscopy and biosensing, and have extensively studied the influence of the position and size of meta-atom hole on the BIC response.^24^ In semiconductor metrology, facile optical measurement on at-resolution structures to determine feature sizes with subnanometer resolution is in high demand, and a seemingly straightforward mechanism based on homogenization has, in fact, many benefits. It promises sensitivity to minute CD differences in patterns with deeply subwavelength-width lines and as a mechanism it is agnostic to the actual CD or pitch chosen. Indeed, the size requirements are on the meta-atom disks and the need to have a somewhat extended array, but not on the nominal periodicity of the actual structure that is subject to inspection. For sensing CD errors that are due to under/over exposure or underetching for instance, one can easily construct convenient readout scenarios on this basis: one can directly compare targets with different feature size (changing the line density, i.e., *N*) and identify if nominally identical fill factors *FF* indeed give identical response (or determine which exposures/etch conditions indeed give nominally identical fill factors *FF*). Additionally, one could envision the currently used library-based approach, which relies on very low contrast differences in spectral measurements, incorporating structure-in-void metasurfaces to take advantage of the strong resonance enhancements. Also, the sensing is to some degree robust: small changes in line orientation, line symmetry, variations in line width along the line length (i.e., CD variations, instead of just CD), or precise placements of the lines in the void will not strongly effect the response. As such it fits precisely the requirements of metrology: determining a single parameter (CD in this case) with maximum precision through prior knowledge (one needs to know the period or the number *N*, and rough line orientation and *FF*), and with maximum robustness against variations in other parameters. This should be contrasted to scenarios in which the task is to obtain a reconstruction with minimal prior knowledge of an unknown object placed in the void, as in an imaging or optical reconstruction task. For such tasks the structure-in-void metasurface approach is quite unsuited: The only sensitivity of the structured void metasurfaces that goes beyond effective index sensing is via the orientation dependence in the effective index mixing rule.

The designs presented here were chosen to combine a practical quality factor *Q*, for presenting a substantial area/volume available for sensing (ca. 35% of the meta-atom), and without any significant optimization efforts already present a simulated sensitivity of 40.5 nm resonant wavelength shift for 1 nm CD change, at 7 nm feature size (chosen as the current litho-node), with an optical line width of 1.8 nm. Experimentally we reached a sensitivity of 3.6 nm wavelength shift per 1 nm CD width change as our feature sizes were around 45 nm, resulting in one optical line width shift for a CD width change of 2.87 nm. We see many opportunities for further optimization. There are many meta-atom designs in the quasi-BIC toolbox where the asymmetry that opens the quasi-BIC coincides with high field confinement in a tight gap,^28^ which can thus embed a deeply subwavelength nanopattern. An interesting question is what design rules optimize sensitivity to particular patterns of interest. In this work we presented simple transmission as a read out mechanism. Instead one could also envision measuring nonlinear light generation, performing polarimetry on scattered light, interferometric readout,^53^ exploring off-normal incidence BICs, or reading out diffraction patterns. In this sense, special targets could be designed to measure alignment errors or astigmatism, or to determine other metrology parameters such as line- or surface roughness and side wall angle. To develop deployable metrology targets and raise the technology-readiness-level of our concept, some key fabrication-line challenges need to be addressed in an industrial setting. These challenges include statistical robustness against wafer-to-wafer variations, resonance drift due to chuck heating, photoresist residue and the influence of the stratified nature of the wafer to spectral behavior. Finally, we notice that in this work we used high-index metasurfaces. An open question is if these methods can also be translated to low-index contrast scenarios. In semiconductor metrology there is a crucial advantage to performing metrology on resists after development yet before etching, as opposed to after etching (when a wafer that does not meet the quality standard can no longer be used). The disk-void metasurface design could serve as a sensor, with resist nanopatterns embedded in the void through a two-step lithography process. While the trade-offs between index contrast, spatial extent of the metasurface, and index sensitivity are not fully explored yet, a promising fact is that the basic mechanism of quasi-BIC formation also operates in low index gratings.

## Methods

### Metasurface Fabrication

A standard e-beam lithography recipe was used to fabricate the nanostructures. First, fused quartz substrates (12 × 12 mm, 500 μm thick, Siegert Wafer GmbH) were cleaned via sonication in water for 10 min, followed by immersion in a base piranha solution at 75 °C for 15 min. The substrates were then dipped in water and rinsed with isopropanol (IPA). We use the same recipe as in the ref 20, however, with two distinct differences. A 75 nm-thick polycrystalline silicon layer was deposited using e-beam evaporation (Polyteknik Flextura M508 E) by heating silicon pellets with an emission current of 90 mA to achieve a deposition rate of 0.1 nm/s. The samples were subsequently subjected to oxygen plasma treatment for 2 min to grow a thin passivation layer, which protected the silicon during development and enhanced adhesion to the resist layer. A ca. 40 nm-thick layer of hydrogen silesquioxane (HSQ) resist (Dow Corning, XR-1541 E-Beam Resist) was spin-coated at 4000 rpm (ramp rate: 1000 rpm/s) for 45 s and baked at 180 °C for 2 min. To mitigate charging effects during patterning, a thin conductive layer of 10 nm aluminum is evaporated by thermal evaporation (Polyteknik Flextura M508 E). This layer of aluminum does not influence patterning properties and is etched away by the resist-developer. Electron beam patterning was performed using Raith’s Voyager system at 50 kV, with an average dose of 1500 μC/cm^2^. The development process began with a 70-s immersion in TMAH at 60 °C, then two subsequent water rinses and one IPA immersion for 15 s each. Finally, the HSQ mask was transferred into the silicon via reactive ion etching (Oxford Instruments Plasma Technologies, Plasmalab 80 Plus) using a CHF_3_/SF_6_/O_2_ gas mixture (15/10/3 sccm), a forward power of 150 W, and a chamber pressure of 7 mTorr, achieving an etch rate of ca. 45 nm/min. Excess resist was not removed, as it neither altered the optical properties nor significantly affected the experimental quality. Metasurface fields of a certain design are ca. 250 × 250 μm, with 100 μm distance to its neighboring field.

### Experimental Transmission Measurements

Metasurface transmission measurements were carried out in a home-build transmission microscope. White light from a halogen source (Avantes, Avalight-HAL, 360–2600 nm) passes through a linear polarizer (Thorlabs) and is loosely focused through the backside of the sample on the metasurface using a 30 mm working distance C-coated lens (Thorlabs). This setup approximates normal incidence excitation, minimizing the broadening of the quasi-BIC resonance caused by multiangle illumination. Transmitted light with an NA of 0.9 is collected using a 100× microscope objective (Nikon, CFI Plan Apo BD) and subsequently focused with an *f* = 50 mm C-coated lens onto a 100 μm core multimode fiber. The sample is illuminated from the back side, as the collection objective has no coverslip aberration correction. Finally, the fiber feeds the collected light into an optical spectrum analyzer (Thorlabs, OSA202C). Reference measurements to generate transmittance spectra are taken adjacent to the metasurface fields.

### Near-Field and Transmission Simulations

We use the RF module in COMSOL Multiphysics 5.2 to numerically solve Maxwell’s equations for a system comprising polycrystalline silicon meta-atoms in air situated on a glass substrate (*n*_air_ = 1, *n*_glass_ = 1.44, *n*_Si_ = 3.45). The meta-atoms are arranged in a square lattice with a pitch of *p* = 955 nm, modeled using periodic boundary conditions to imitate an infinite extended array in the *xy*-plane. Void structures are parametrized using a fill factor, which scales the radius of azimuthal features or angular regions in the case of radial designs. This enables a parameter sweep over the fill factor combined with a frequency sweep, sampling 200 frequency points. Perfectly matched layers (PML) are implemented above and below the meta-atom plane along the *z*-axis, positioned at least twice the wavelength away from the structure, to effectively absorb outgoing scattered light. The system includes an input port and an output port defined at the interfaces of the glass and air regions with the PMLs, respectively. Transmission spectra are calculated by evaluating the transmitted energy flow from input port (in the glass) to output port (in air). The meta-atoms are illuminated from the glass side with an incident field polarized in the vertical direction. This polarization aligns perpendicular to the asymmetry of the meta-atoms, which is oriented horizontally.

## Supplementary Material



## Notes

I. Fill factor estimation and image analysis procedure; II. All experimental transmittance spectra for different structural infilling designs; III. Finite-sized array simulations (PDF)
