# Supplementary material for: Structure-in-Void Quasi-Bound State in the Continuum Metasurface for Deeply Subwavelength Nanostructure Metrology
Source: ACS Nano. 2025 Sep 2;19(36):32082–92. doi: 10.1021/acsnano.5c03366 (PMC12444989; doi:10.1021/acsnano.5c03366)
Supplement: Supplementary file 1 [file nn5c03366_si_001.pdf]

Supporting Information:

Structure-in-Void Quasi-Bound State in the Continuum Metasurface for Deeply Subwavelength Nanostructure Metrology

Falco Bijloo,[1,2*] Arie J. den Boef,[1,3,4] Peter M. Kraus,[1] and A. Femius Koenderink[2*]

[1]Advanced Research Center for Nanolithography,

Science Park 106, 1098 XG Amsterdam, The Netherlands

[2]Department of Physics of Information in Matter and Center for Nanophotonics,

NWO-I Institute AMOLF, Science Park 104, 1098 XG Amsterdam, The Netherlands

[3]Department of Physics and Astronomy, and LaserLaB,

Vrije Universiteit, 1081 HV Amsterdam, The Netherlands

[4]ASML Netherlands B.V., 5504 DR Veldhoven, The Netherlands

[*]To whom correspondence should be addressed; E-mail: bijloo@amolf.nl; koenderink@amolf.nl

(Dated: July 14, 2025)

I. FILL FACTOR ESTIMATION AND IMAGE ANALYSIS PROCEDURE

The fill factor (FF) is estimated by using a thresholding procedure. In essence, this comes down to a script that reads a TIFF file that contains a SEM image of a structured void metasurface.

Figure S1.1 shows a sequence of images, starting with a raw SEM image, and leading to the isolation of a single meta-atom, from which we determine fill factor. First, the contours in the metasurface SEM images are found by using an adaptive thresholding called cv2.adaptiveThreshold by OpenCV (version 4.9.0, Python version 3.9.13). This method uses small groups of neighboring pixels to find an optimal threshold value for each neighbor. Second, a circle is fitted to the image to find a disk radius and center. This is used as a mask of value 1 and multiplied with the original image, to extract only one meta-atom.

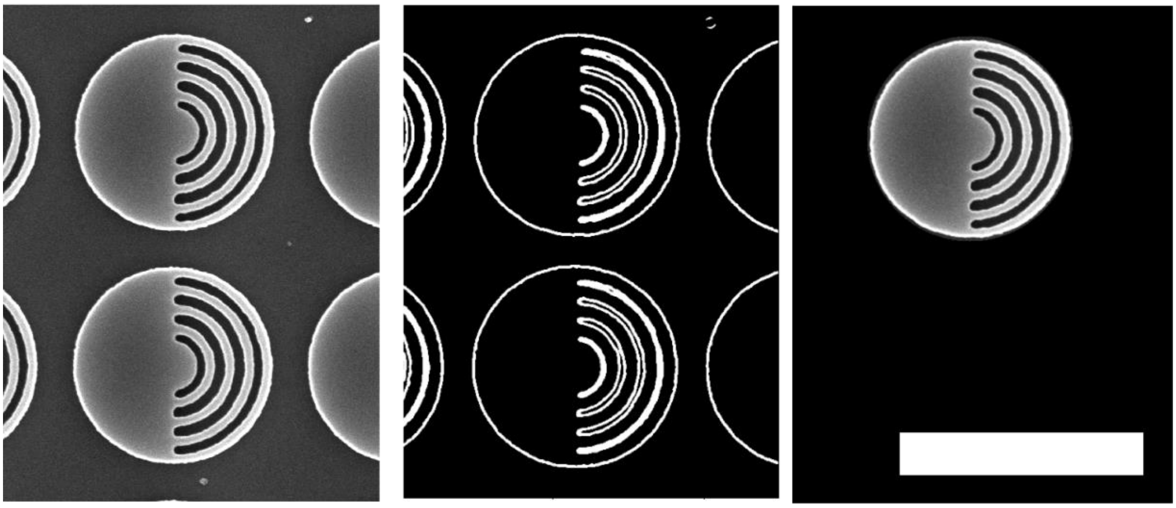

Figure S1.1: Illustration of procedure to isolate a single meta-atom for image analysis of its fill factor. Left: raw SEM image. Middle: boundaries identified by OpenCV. Right. Single meta-atom isolated by fitting a meta-atom outer boundary in the middle image, and masking. The scale bar in the right image denotes 1 μm.

Next, for an isolated meta-atom we determine the fill factor by binarizing. Grey value pixels from the SEM image are binarized into a black and white (0 or 1) image by thresholding. The threshold value needs to be adjusted depending on the image, as many boundaries light up in SEM images. This means that the threshold value is determined per category of structured infillings. We found it judicious to decrease the threshold by 6 points per upwards step in the patterned fill factor for each structure stripe, accounting for the fact that features darken in SEM when boundaries are further apart.The tabulated base threshold values were chosen as follows for the lowest fill factors (A# indicating “Azimuthal with N=#, and R/Pil indicating Radial and Pillar structures):

A1	A2	A3	R2	R8	Pil
100	100	100	100	80	100

The result of such a procedure are shown in Figure S2.

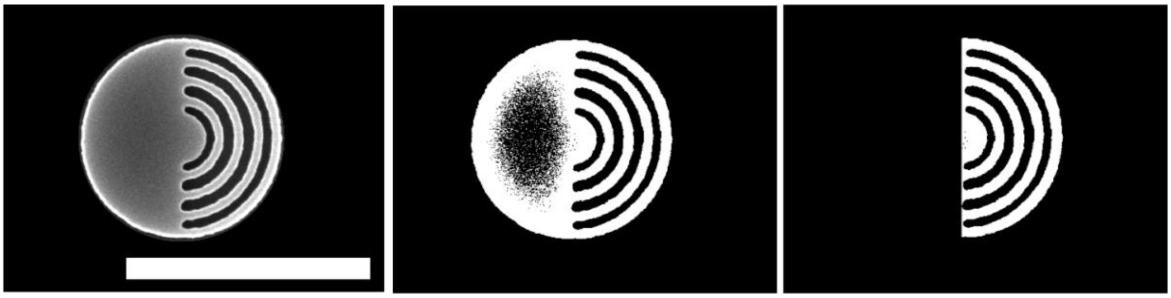

Figure S1.2: Illustration of procedure to determine fill factor. Left: single meta-atom isolated by image analysis (replicated from Fig. S1), scale bar denotes 1 μm. Middle: thresholded image. Right. Clip out for just the structured void part. The FF is determined by counting the fraction of white pixels in the semicircle.

Part of the large surface area on the unstructured left side is considered below the threshold value, even though it is `filled’, an illustration of the statement that areas darken as they are

further away from boundaries. A typical example of this is shown in the middle image in Fig. S1.2. As we are interested only in the structured void part, we cut out only the right half of the circle. Next we count the number of white pixels, which we idenfity as silicon. To convert that into a FF ,we need to ratio to the `fillable area', meaning we need to determine the area of the unfilled void. To do so we examine SEM images for the FF=0 and FF=1 case with the same procedure. This provides the normalization to obtain a FF between 0 and 1, where FF=0 means the same amount of white pixels as the left image of Fig. S3, and FF=1 means the same amount of white pixels as the right image of Fig. S1.3.

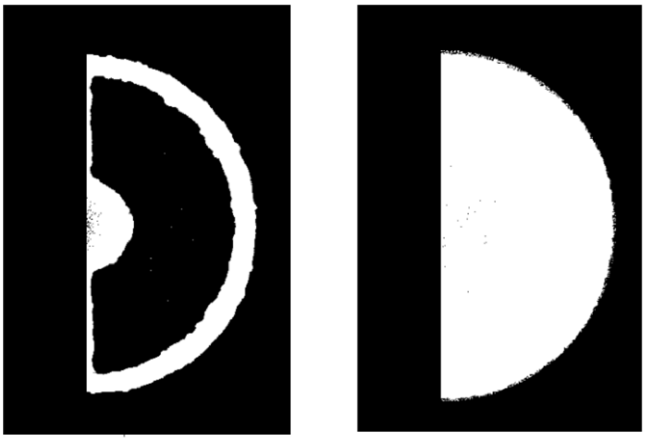

Figure S1.3: Thresholded image for an FF=0 and FF=1 structure. These images are used to determine the area of the void, which is required to normalize to when determining the FF of structured voids.

The thresholding procedure introduces errors. In particular, the extracted FF values depend somewhat on the chosen threshold value. We note that decreasing thresholding values leads to systematic upward (approximately linear) shifts of extracted FF values. Therefore the qualitative behavior of wavelength shift versus FF value changes does not change with thresholding, although the thresholding procedure may introduce a small systematic error in FF.

II. ALL TRANSMITTANCE SPECTRA

In Figure S2.1, we present all experimental transmittance spectra (x-axes) as function of fill factor (y-axis) for all designs. Top left: Azimuthal N=1, top right: azimuthal N=2, middle left: azimuthal N=3, middle right: radial N=2, bottom left: radial N=8, bottom right: Pillars.

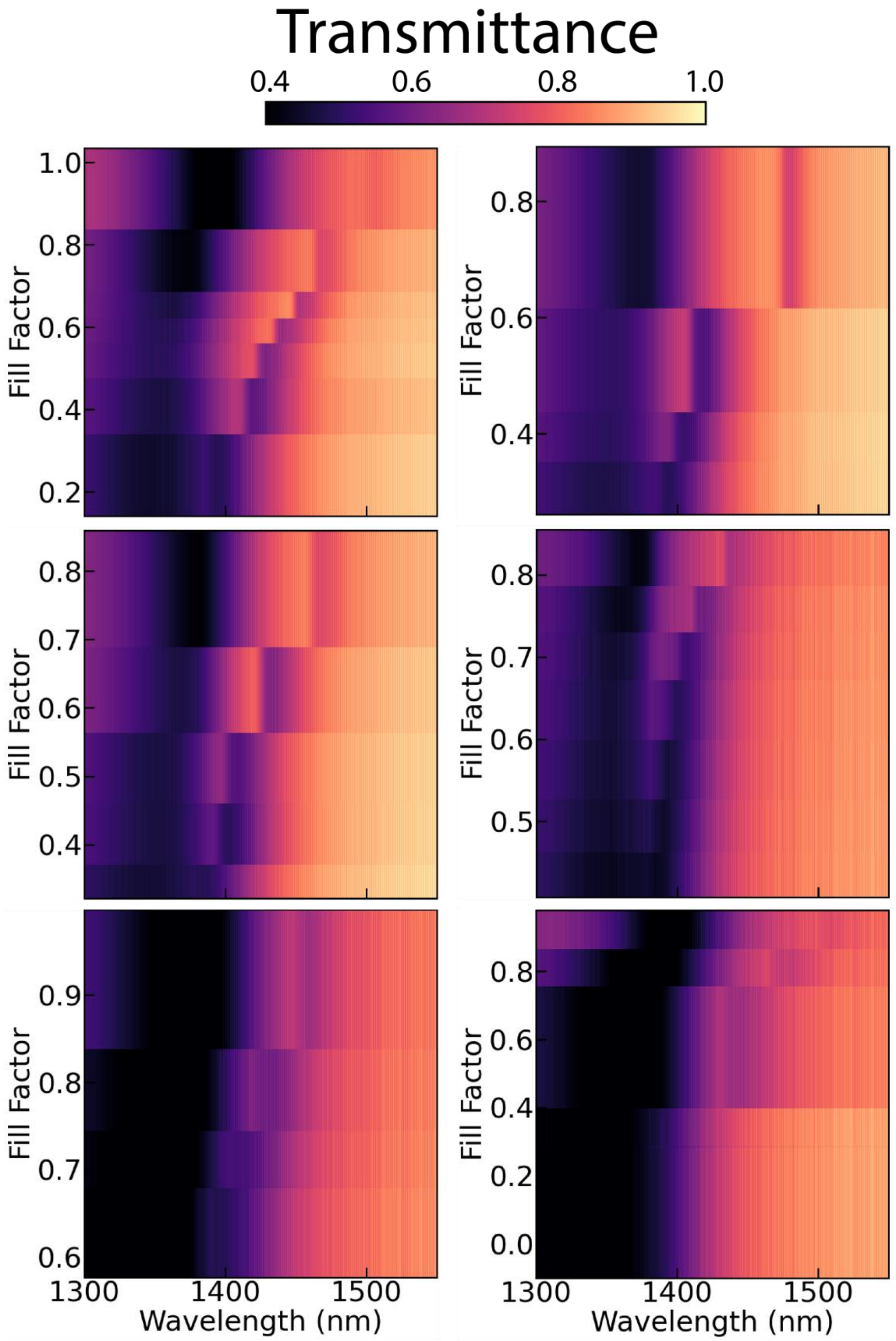

Figure S2.1: Experimental transmittance spectra for all fabricated structured-void metasurfaces. Wavelength is plotted on the x-axes in nm, fill factor FF on the y-axes and transmittance is represented by color, which is denoted by the colorbar on the top. Top left: Azimuthal N=1, top right: azimuthal N=2, middle left: azimuthal N=3, middle right: radial N=2, bottom left: radial N=8, bottom right: Pillars.

III. FINITE SIZED ARRAY SIMULATIONS

To assess whether a low quality factor (Q) arising from finite-sized metasurface arrays (1) still permits informative critical dimension (CD) measurements, and (2) is primarily limited by measurement process or by the device footprint, we performed finite element simulations using JCMSuite. The simulations were performed using both the scattering solver and the open system eigenmode solver. The simulated systems all consist of a glass substrate and an air superstrate, both 900 nm thick, with the metasurface being 60 nm thick. Each meta-atom has a radius of 398 nm, and the meta-atoms are arranged in a square lattice with a lattice vector length of 953 nm. The mesh of the periodic simulations has a maximum side length of 175 nm in the substrate and superstrate, and 44 nm in the metasurface. The mesh of the 20x20 finite array simulations has a maximum side length of 233 nm in the substrate and superstrate, and 59 nm in the metasurface. The simulation software uses open PML boundary conditions which are automatically generated.

First, periodic structures were simulated to benchmark against our COMSOL simulations. We observed similar resonance behavior in both methods for the case of an N=3 azimuthal concentric grating with fill factor (FF) = 0.55. The magnetic dipole mode is clearly identified and visualized in Fig. S3.1, where the magnetic field |H| shows a strong spot which is pointing out of the plane (H ~ Hz in this case) and the electric field |E| shows the typical ring.

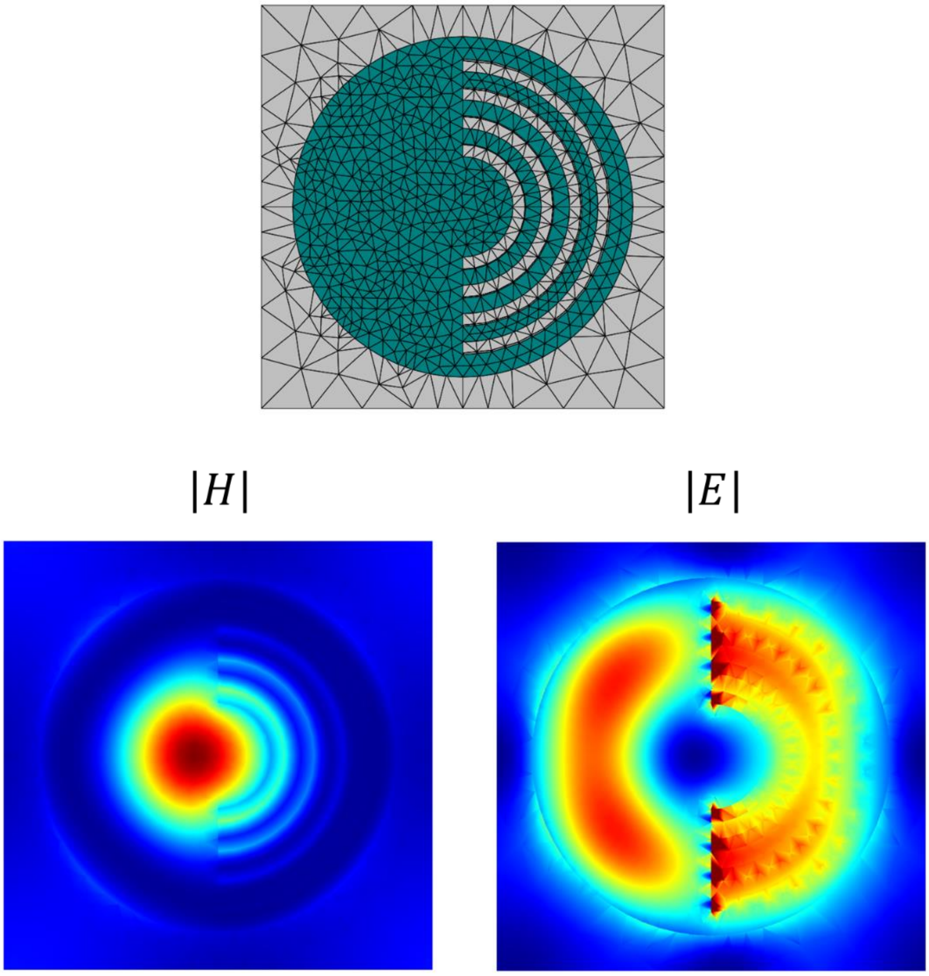

Figure S3.1: Top: Mesh of the periodic simulations. Bottom: Absolute value of the H- and E-fields of the eigenmode of a single unit cell of the metasurface, with periodic boundary conditions in the X,-X,Y,-Y directions and transparent PML boundary conditions in the Z,-Z directions. The magnetic dipole mode is clearly visible. The eigenmode has a frequency of 1331.1 - 1.9002i THz. All calculations were performed using JCM, with finite element degree equal to 2, resulting in around 3x105 elements, 10 GB memory use, and a solving time of about 1 min on a typical PC.

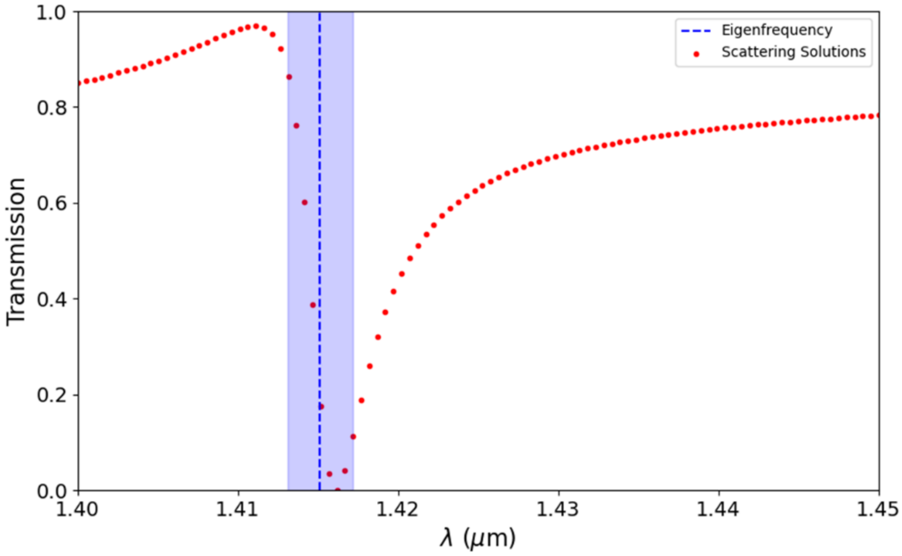

Figure S3.2: Transmission spectrum of the periodic metasurface around the Fano resonance. Every dot corresponds to the result of a scattering simulation. The vertical dashed line indicated the resonant wavelength as predicted by the eigensolver, and the shaded area around the resonant wavelength indicated the linewidth as predicted by the imaginary part of the eigenfrequency. We see a clear Fano lineshape, and a good correspondence between the scattering results and the predictions of the eigenmode approach. All calculations were performed using JCM, with finite element degree equal to 2. The eigenmode solution is identical to that of figure S3.1, and each of the 100 scattering simulations requires around $3x10^5$ elements, 5 GB memory use, and a solving time of about 22 sec on a typical PC.

Finite sized array simulations for 20x20 unit-cells at FF = 0.55, reveal through eigenmode analysis a similar magnetic dipole mode, which is visualized in Fig. S3.3. The magnetic dipole mode is clearly visible as strong spots in the magnetic field H and circles in the electric field E, which are similar to the periodic analysis. In the plane of the metasurface, at its edge, we add a buffer of one unit cell thick around the metasurface, and the computation is made more efficient by adding a perfect electric conductor symmetry plane in the -Y direction, splitting the sample horizontally. We found an eigenfrequency of 1328.4 - 7.6378i THz, corresponding to a resonant wavelength of 1418 nm, with an expected linewidth of 16 nm and a quality factor Q ~ 87. Via Fig. 2b in the manuscript, we extrapolate this footprint-limited quality factor for an N=20 concentric grating metasurface, which shows that in a practical CD metrology application, still ~0.5nm CD change results in a single optical linewidth shift.

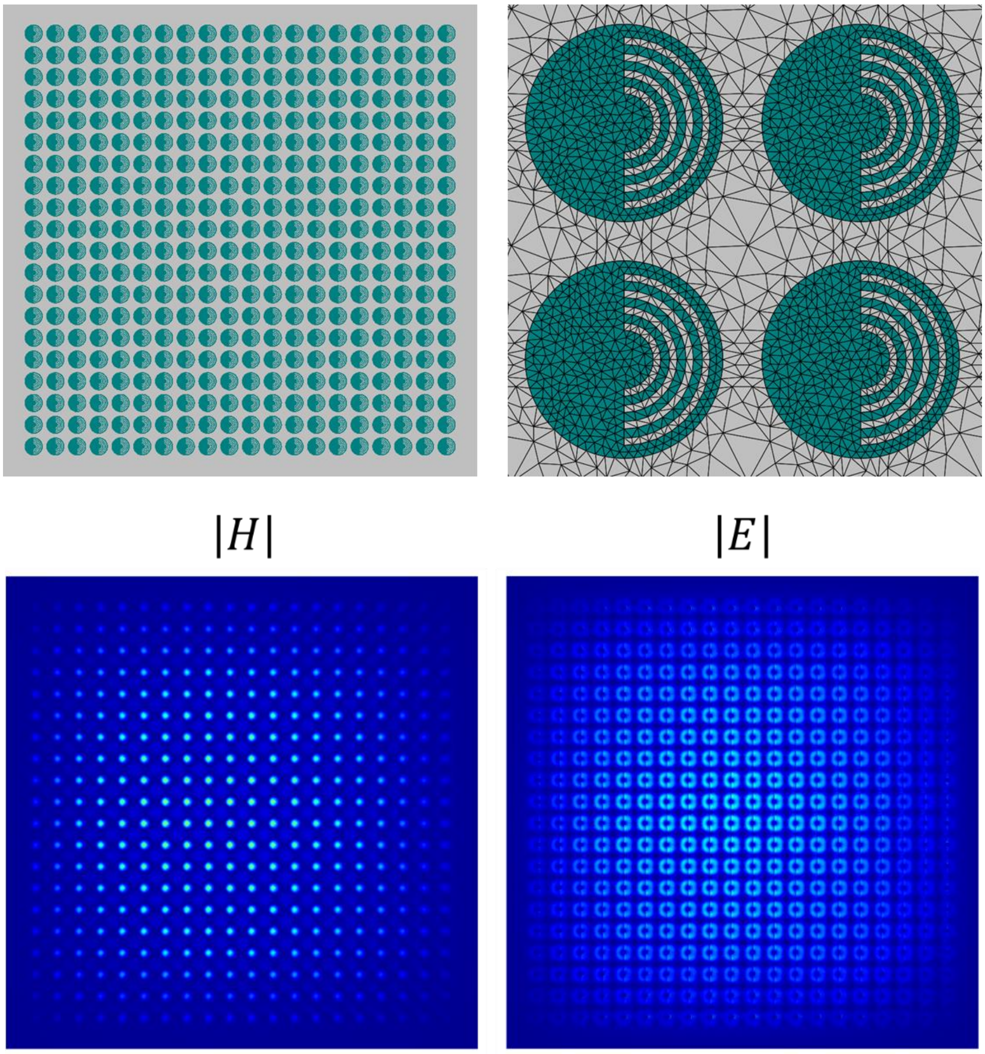

Figure S3.3: Top: The left image shows the 20x20 finite size array of the metasurface, while the right image shows the mesh used for the simulation, zoomed in on four meta-atoms. Bottom: The absolute value of the H- and E-fields of the eigenmode of a 20x20 finite sized array of the metasurface in rainbow colormap blue (min) to red (max), with transparent PML boundary conditions in every direction except the -Y direction, which defines a perfect electric conductor symmetry plane. The eigenmode has a frequency of 1328.4 – 7.6378i THz. All calculations were performed using JCM, with finite element degree equal to 1, resulting in around $6x10^6$ elements, 110 GB memory use, and a solving time of about 1 hour on a typical PC.
